# Testing a Web-Based Interactive Comic Tool to Decrease Obesity Risk Among Racial and Ethnic Minority Preadolescents: Randomized Controlled Trial

**DOI:** 10.2196/58460

**Published:** 2025-01-15

**Authors:** May May Leung, Katrina F Mateo, Marlo Dublin, Laura Harrison, Sandra Verdaguer, Katarzyna Wyka

**Affiliations:** 1 Friedman School of Nutrition Science and Policy Tufts University Boston, MA United States; 2 Graduate School of Public Health Policy, City University of New York New York, NY United States; 3 School of Urban Public Health, Hunter College, City University of New York New York, NY United States

**Keywords:** childhood obesity, preadolescents, racial and ethnic minority populations, dietary behaviors, BMI, digital health

## Abstract

**Background:**

Childhood obesity prevalence remains high, especially in racial and ethnic minority populations with low incomes. This epidemic is attributed to various dietary behaviors, including increased consumption of energy-dense foods and sugary beverages and decreased intake of fruits and vegetables. Interactive, technology-based approaches are emerging as promising tools to support health behavior changes.

**Objective:**

This study aimed to assess the feasibility and acceptability of Intervention INC (Interactive Nutrition Comics for Urban, Minority Preadolescents), a 6-chapter web-based interactive nutrition comic tool. Its preliminary effectiveness on diet-related psychosocial variables and behaviors was also explored.

**Methods:**

A total of 89 Black or African American and Hispanic preadolescents with a mean age of 10.4 (SD 1.0) years from New York City participated in a pilot 2-group randomized study, comprising a 6-week intervention and a 3-month follow-up (T4) period. Of the 89 participants, 61% were female, 62% were Black, 42% were Hispanic, 53% were overweight or obese, and 34% had an annual household income of <US $20,000. Participants were randomly assigned to the experimental group (45/89, 50% received the web-based comic tool), or the comparison group (44/89, 50% received web-based nutrition newsletters). Primary measures included feasibility and usability at intervention midpoint (T2) and intervention end (T3). Semistructured interviews were conducted at the same time to assess acceptability and satisfaction. Secondary measures, collected at baseline (T1), T2, T3, and at T4, included attitudes, beliefs, and behaviors related to fruit, vegetable, water, sugar, and junk food intake. Descriptive analyses were conducted for use and usability data. Interviews were systematically analyzed to facilitate identification of patterns and themes. Secondary data were analyzed using descriptive statistics. Within- and between-group effect sizes were reported.

**Results:**

In total, 72% (33/45) and 60% (27/44) of the experimental and comparison groups, respectively, accessed their tool weekly. The mean total usability score was high and moderately high for the experimental and comparison groups, respectively (mean 4.01, SD 0.37 and mean 3.81, SD 0.51; *P*=.048), based on a 5-point Likert scale). Children in both groups found the tool acceptable, and few reported difficulties logging in or accessing content. Between-group effect sizes for beliefs and attitudes related to dietary intake, while favoring the experimental group at T3, were in the small range. These improvements in both groups were largely diminished by T4. However, between-group effect sizes for behaviors related to fruit, vegetable, and water intake, favoring the experimental group, were medium to large and were maintained at T4.

**Conclusions:**

This pilot feasibility study suggests that an interactive comic tool may be an appealing and useful format to promote positive dietary behaviors in racial and ethnic minority preadolescents. However, further research, including a full-scale randomized controlled trial, is warranted to determine the effectiveness of Intervention INC.

**Trial Registration:**

ClinicalTrials.gov NCT03165474; https://clinicaltrials.gov/study/NCT03165474

**International Registered Report Identifier (IRRID):**

RR2-10.2196/10682

## Introduction

### Background

The prevalence of childhood obesity in the United States, especially among racial and ethnic minority communities with low incomes, remains consistently high and shows no signs of stabilizing. Specifically, Hispanic and Black or African American children have markedly higher rates of obesity (25.8% and 22%, respectively) compared to the national prevalence of 18.5% [1], with rates rising as age progresses, especially in African American and Hispanic children [2]. Compared to school-age children (18.4%) and preschoolers (13.9%), adolescents (20.6%) have a higher prevalence of obesity [3,4]. In the short term, obesity in youth may lead to issues such as an increased risk of elevated blood pressure and lipid concentrations, anxiety, and depression [5,6]. In the long term, adverse health conditions, including cardiovascular disease, diabetes, and disabilities, can manifest in youth who are obese and be maintained into adulthood [7-10].

Childhood obesity has been linked to several behavioral determinants, namely, increased consumption of highly processed, energy-dense foods and sugary beverages and a decreased intake of fruits and vegetables [11-13]. With the pervasiveness of daily technology and new media use in youth, particularly within the Hispanic and African American population [14], digital platforms are increasingly being used to deliver creative messaging around health and nutrition to children and their caregivers [6]. These tools, which include mobile apps and interactive websites, are emerging as promising approaches to engage and deliver tailored interventions to support health behavior change [15]. However, knowledge gaps in the usability, engagement, and usefulness of such applications still exist, limiting their potential effectiveness [16]. Furthermore, there is a shortage of effective health promotion interventions that are culturally tailored to address the needs and preferences of populations with disproportionately high rates of chronic disease. Even fewer interventions have been designed specifically for children, particularly those transitioning from childhood to adolescence [17]. It is crucial to intervene during this development stage as food preferences established during this period often persist into adulthood [18-20]. In addition, children at this age are gaining autonomy and developing decision-making skills related to dietary behaviors [21], which highlights the importance of intervening at this critical period.

### Study Purpose

The primary purpose of this pilot study was to assess the feasibility and participant acceptability of Intervention INC (Interactive Nutrition Comics for Urban, Minority Preadolescents), an interactive, web-based health promotion tool. In addition, this study aimed to explore tool effectiveness by determining if it improved attitudes, beliefs, and behaviors related to obesity. Taken together, these data will provide insight for further improvements to the tool and reveal user preferences, which is important feedback for developing culturally tailored, effective, and engaging digital tools, particularly for populations at risk for childhood obesity.

## Methods

### Study Design

The Intervention INC study was a pilot, 2-group randomized controlled trial (RCT) that evaluated a 6-week intervention promoting fruit, vegetable and water consumption, with a 3-month follow-up (T4) period. A protocol for this feasibility trial was published and registered with the Clinical Trials Registry (NCT03165474) [22]. Parent-child dyads were randomized into either the experimental or comparison group, with both child and parent participants blinded to their assigned group. Details regarding the randomization process are provided in the protocol paper [22].

### Participants

Children residing in New York City (NYC) were recruited based on the following inclusion and exclusion criteria: self-identified as Black, African American, or Latino; aged between 9 and 12 years (preadolescents) at the time of scheduled baseline (T1) visit; read and spoke in English; had a BMI percentile ≥5% at T1 (categorized as healthy, overweight, or obese); had regular internet access via a tablet device, mobile phone, or computer or laptop; had regular access to a phone with texting capability; was comfortable reading or viewing material on electronic devices; was comfortable speaking with study staff about thoughts or experiences while participating in the study; had no allergies, food aversions, food disorders, or medications with side effects that may impact participation in the study; did not have a pacemaker or heart condition; and had a legal parent or guardian willing to participate in the study. While parents or guardians were a part of this study, the focus of this paper is to present primary outcome data for the child participants.

Multiple recruitment approaches were implemented between August and November 2017. Initially, mailings were sent to parents or guardians of potentially eligible child patients of a community-based clinic (partnering organization) in Harlem, NYC. Recruitment efforts were expanded to include community flyers in East Harlem and Upper Manhattan as well as posting notices in and around local businesses, housing complexes, community centers, schools, and churches. Through partnerships with local schools and community initiatives, recruitment efforts also occurred via tabling at community and school events. Further details regarding study recruitment and sample size considerations are included in other publications [22,23].

### Intervention

Content for the child participants, regardless of group assignment, was tailored (to include more information related to either fruit and vegetable or water) based on responses to initial screening questions, at T1, related to child fruit, vegetable and water intake, child self-efficacy to increase fruit, vegetable and water intake, and parent self-efficacy to support the child in increasing fruit, vegetable and water intake.

#### Experimental Group

Children in the experimental group received access to Intervention INC, an interactive web-based comic tool informed by the Health Belief Model [24], Social Cognitive Theory [25], and the Narrative Transportation Theory [26], which contained health messages focused on fruit, vegetable and water consumption. It comprises a 6-chapter comic with pop-up windows (highlighting health facts, food-related fun facts, or character information) and prompted audio and visual effects to enhance tool engagement. It also includes a weekly goal setting and assessment feature and texting or email messages reminding children to read the comic and work on their selected health behavior goals. Most of the health messages are delivered by the comic characters themselves in a narrative form, but some messages were delivered didactically (such as in the pop-up windows), as entertainment education research suggests a combination of narrative and nonnarrative information may be the most effective in improving health beliefs and behaviors [27]. Multiple behavior change techniques (BCTs) were used to promote healthy dietary behaviors. Table 1 lists all the BCTs that were incorporated into the comic and examples of how each of them were used. The comic tool was designed and developed by a collaborative research team, consisting of scientists, digital health experts, content and system developers, and intended users (Black or African American and Hispanic preadolescents and their parents). Extensive formative research, including usability testing, was conducted during the development process [28]. Additional details of the intervention, including its development and specific components, have been described in previous papers [22,28].

**Table 1:** BCTs^a^ incorporated into the web-based comic tool, Intervention INC (Interactive Nutrition Comics for Urban, Minority Preadolescents).

BCT	Action	Example
Goal setting (behavior)	At the end of each comic chapter, a character prompts the child to select a weekly goal to work on.	End of chapter message includes a picture of a character along with a prompt to set a goal: “Let’s pick a goal to work on for the week!” Sample goal: “I will eat fruits I like (such as bananas or apples) at breakfast.”
Self-monitoring of behavior	The following week, the child is prompted to assess how they did with the weekly goal they selected to work on.	End of chapter message: “Your goal for last week was (selected goal).” “How often did you do this in the last week?” Response options: never, sometimes, most of the time, and all of the time
Feedback on behavior	On the basis of the child’s self-assessment of the weekly goal, feedback is provided on the performance of the behavior.	Sample message if a child achieves the goal: “Congrats! Keep up the good work!” Sample message if a child does not achieve the goal: “That’s ok! Michael Jordan was cut from his high school basketball team, and he didn’t give up!”
Social support	Children can select weekly goals related to practical social support from their parents.	Sample goal related to practical parental social support: “I will ask my parent to pack a water bottle for me when I leave the house this week.”
Information about health consequences	Information about the consequences of eating healthfully or unhealthfully is provided in the comic in didactic and narrative forms, such as pop-up messages, character dialogue, and end of chapter messages from characters.	Sample pop-up message (in a panel where one character tells another to stay hydrated): “Did you know that not having enough water can make you feel dehydrated? Grab a water if you’re feeling tired or you're having a hard time concentrating.”
Salience of consequences	Scenes are included in the comic to specifically emphasize the consequences of performing both positive and negative dietary behaviors, with the aim of making them more memorable.	A meter highlights the energy levels of the comic characters based on the nutritional quality of the foods they consume. For example, a character eats a banana, and his energy meter rises. He also acknowledges how “great” he feels.
Demonstration of behavior	Comic characters model positive dietary behaviors.	The father of the main character is seen drinking water and eating fruits and vegetables in multiple scenes.
Prompts or cues	Messages from the comic characters are delivered weekly (via texting, email, or both) to remind children to work on the goal they selected that week.	Sample text or email message from a character: “Hey, [child name]—We have a mission for you! Be sure to log in and choose a goal if you haven't already. Then you’ll get tips to train and get stronger! See you soon!”
Social reward	Positive feedback is provided if the child achieves their weekly goal.	Bonus content related to the characters and comic story is released if the child achieves their weekly goal.

^a^BCT: behavior changes technique.

#### Comparison Group

Children in the comparison group received access to 6 web-based newsletters. Health-related content for the comparison group was similar to that for the experimental group but presented in a newsletter (didactic) format, which includes tips, recipes, diet-related knowledge and facts, and web-based games. Similar to the experimental group, the comparison group also had a weekly goal setting and assessment component and received reminder messages via texting, email or both.

### Data Collection and Measures

#### Overview

Data were collected at 4 different time points: T1, intervention midpoint or 3 weeks after T1 (T2), intervention end or 6 weeks after T1 (T3), and T4. Brief descriptions of our primary and secondary outcome measures are provided in the following sections, while details of all measures are described elsewhere [22]. T1 and T4 data collection was conducted in person at the study site, while T2 and T3 data collection was conducted via phone. At T2 and T3, links to any questionnaires were sent to the participants ahead of time to complete, and then, responses were confirmed during the phone call. If the questionnaire was not completed before the call, children completed it during the call with a trained research assistant. Table 2 lists the measures collected and the time points at which they were collected.

**Table 2:** Key primary and secondary measures, data sources, and time points at which the measures were assessed.

Measures		Data source	Time points				
			T1^a^	T2^b^	T3^c^	T4^d^	O^e^
**Feasibility and acceptability measures (primary measures)**							
	Use of the web-based tool	Tracking system (internally created)					✓
	Usability of the web-based tool	Interview and questionnaire items^f^		✓	✓	✓	
	Acceptance and satisfaction	Interview				✓	
**Outcome measures (secondary measures)**							
	Dietary beliefs and attitudes	Questionnaire items^f^	✓	✓	✓	✓	
	Dietary intake	Questionnaire items^f^	✓	✓	✓	✓	
	BMI-for-age percentile	Digital stadiometer	✓			✓	

^a^T1: baseline.

^b^T2: midpoint (3 weeks after baseline).

^c^T3: intervention end (6 weeks after baseline).

^d^T4: follow-up (3 months after intervention).

^e^O: ongoing throughout the intervention period.

^f^All questionnaire items were either taken from or directly informed by validated questionnaires.

#### Feasibility and Acceptability Measures (Primary Measures)

##### Use of the Web-Based Comic Tool

Use data, such as the number of weeks logged in, were captured throughout the intervention on a custom-built platform. Additional use data related to goal setting were collected, including the percentage of participants who selected, assessed (ie, “How often did you do this [behavior] in the last week?”), and achieved (defined as responses of “Most of the Time” or “All the time”) weekly dietary goals.

##### Usability of the Web-Based Comic Tool

Five domains of usability (usability, usefulness, ease of use, ease of learning, and satisfaction) were assessed with a 30-item questionnaire on a 5-point Likert scale (ranging from strongly disagree to strongly agree). This questionnaire was adapted from the System Usability Scale [29]; Usefulness, Satisfaction, and Ease-of-use questionnaire [30]; and an acceptability or usability measure by Ben-Zeev et al [31]. Minor modifications were made to tailor the questionnaire to the literacy levels of our intended population based on pilot testing with them. Specific details related to the modification process are described elsewhere [22]. The modified questionnaire was not validated for web use. A total score of 4.0 out of 5.0 was considered high usability.

##### Acceptance and Satisfaction

Semistructured interviews were conducted at T2 and T3 where open-ended questions were asked to both experimental and comparison group participants about the experience of engaging with their web-based tool. Specifically, interview questions aimed to assess participant acceptability and satisfaction with the overall tool and its specific components.

#### Outcome Measures (Secondary Measures)

##### Dietary Beliefs and Attitudes

Outcome expectations (OEs), self-efficacy, behavioral intention (BI), and attitudes related to fruit, vegetable, water, junk food, and sugary drinks consumption were assessed at all 4 time points with an 84-item questionnaire, which was informed by and modified from the validated ProChildren Questionnaire and the validated Reynolds Questionnaire [32,33]. The OE, self-efficacy, BI, and attitudes questionnaires had a Cronbach α of 0.87, 0.74, 0.52, and 0.70, respectively.

##### Dietary Intake

Frequency of consumption of fruit, vegetable, water, junk food and sugary drinks during the past 7 days were assessed at all 4 time points with a 17-item questionnaire comprising questions from and informed by the validated 2017 Youth Risk Behavior Surveillance System questionnaire and the validated Beverage and Snack Questionnaire [34,35]. Three items assessing the intake of different types of water were internally created. The dietary intake questionnaire had a Cronbach α of 0.79.

##### BMI-for-Age Percentile

The height and weight of child participants were measured at T1 and T4 using standardized methods [36]. A SECA 264 digital stadiometer and Tanita MC-780U body composition monitor were used to collect anthropometric measures. The Centers for Disease Control and Prevention BMI percentile calculator was used to determine BMI-for-age percentile [37].

### Data Analysis

#### Feasibility and Acceptability Measures (Primary Measures)

##### Use and Usability of the Web-Based Comic Tool

Use of the web-based tool for each group was assessed by first aggregating the “click-tracking” data by participant, URL, study week, and across the intervention period. Descriptive analyses, including means, SD, and ranges (minimum-maximum) were performed. Similar descriptive analyses were also conducted to measure the overall score for the usability data of the web-based tools across both groups.

##### Acceptance and Satisfaction

Interviews from T2 and T3 were systematically analyzed by 3 trained coders through a streamlined process of directly listening to individual audio files and then transferring relevant information into a matrix sheet to produce a summary of overall impressions of usability, acceptability, and feasibility of the tool and its specific components (ie, reminders and goal setting). A rapid memo technique, informed by the Rapid Evaluation and Assessment Method [38] and the Rapid Identification of Themes From Audio Recordings [38] analysis method, was used to summarize interview responses as well as to note key quotations. These techniques have been used as a way to preclude the traditional steps of transcribing qualitative data and coding transcripts when resources are limited and quick reporting time is needed [38,39].

Audio files were randomly assigned to each coder. In addition to the primary coder, a secondary coder reviewed the audio files for accuracy, noting any additional information, until very few additions were being made or discrepancies were noted. This process was used for approximately the first 10 interviews. However, when a coder had a subsequent audio file with inaudible sections, another coder conducted a secondary review and added any missing information to the matrix. Coders met frequently throughout the analysis process to compare their results; discuss overall matrix content and trends; identify, clarify, and understand emergent findings; and highlight representative quotations.

#### Outcome Measures (Secondary Measures)

Child and parent sociodemographic characteristics were compared between conditions (experimental or comparison) using 2-tailed independent samples *t* tests and chi-square tests. To assess improvements in outcome measures (child dietary beliefs and attitudes, dietary intake, and BMI-for-age percentile), change scores (relative to T1) were computed and summarized using descriptive statistics. To estimate the magnitude of the effects at T3 and T4, both within- and between-group effect sizes were computed on change scores. Analyses were completed using SPSS statistical software (version 26; IBM Corp) [40]. Of note, the data analyst was blinded to the intervention assignment to minimize bias.

### Ethical Considerations

All study activities were approved by the Institutional Review Board at Hunter College in New York, New York (2015-0547). Participation was entirely voluntary, and study participants could choose to withdraw from the study, without any reason, at any point in time during the study. Informed consent, child assent and parental permission were obtained before study participation. Both child and parent participants were compensated. Children received up to US $70 and parents or guardians received up to US $65 (in the form of gift cards) for completing data collection. To maintain participant privacy and confidentiality, all study data have been deidentified.

## Results

### Participants

**Figure 1:**
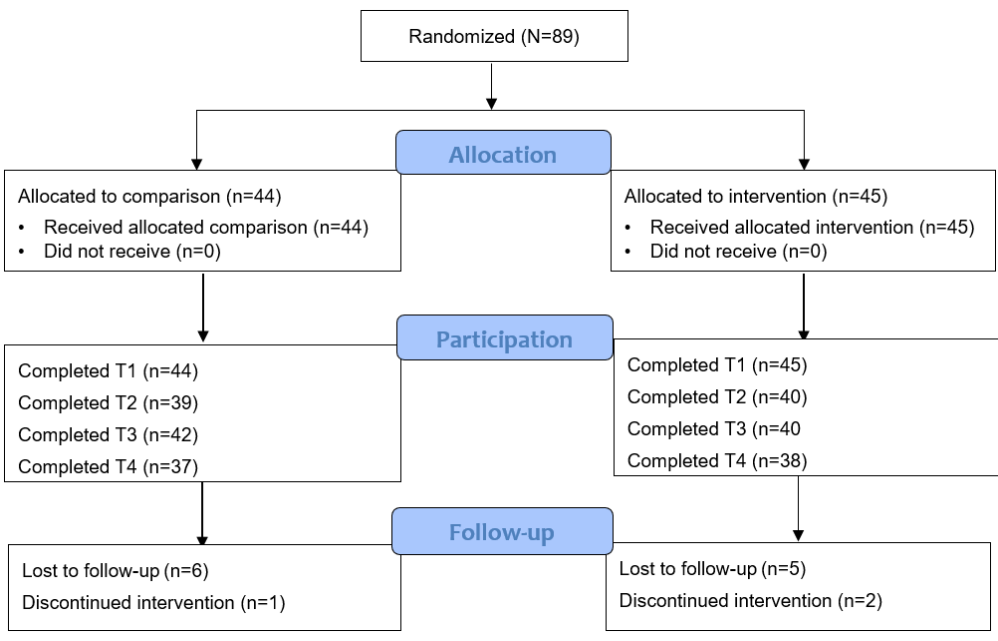
CONSORT (Consolidated Standards of Reporting Trials) diagram of the randomization and retention progress throughout the study. T1: baseline; T2: midpoint (3 weeks after baseline); T3: intervention end (6 weeks after baseline); T4: follow-up (3 months after intervention).

A total of 89 child-parent dyads were recruited and completed T1 measures. There were no differences in characteristics between the 2 groups. The mean age of child participants was 10.38 (SD 1.03) years; the majority were female (54/89, 61%), Black or African American (42/89, 47%) or Hispanic (29/89, 33%), and overweight or obese (47/89, 53%). The mean age of parent participants was 40.83 (SD 8.87) years; nearly all were female (84/89, 94%), born in the United States (63/89, 71%), with some college education or more (61/89, 68%), and single (40/89, 45%). One-third (30/89, 34%) of the dyads reported an annual household income of <US $20,000, and nearly two-thirds (54/89, 61%) reported receiving Supplemental Nutrition Assistance Program benefits. Refer to Table 3 for additional demographic details. The CONSORT (Consolidated Standards of Reporting Trials) diagram (Figure 1) shows the randomization and retention progress throughout the study. Retention rates were high: T2, 87% (77/89); T3, 89% (79/89); and T4, 84% (75/89) [23].

**Table 3:** Baseline child and parent demographic characteristics (N=89).

Characteristics				Overall (N=89)		E^a^ (n=45)		C^b^ (n=44)		*P* value^c^					
**Child**															
	Age (y), mean (SD)		10.38 (1.03)		10.38 (1.06)		10.39 (1.01)		.97						
	**Sex n (%)**														
		Male	35 (39)		17 (38)		18 (41)		.76						
		Female	54 (61)		28 (62)		26 (59)		—^d^						
	**Race or ethnicity, n (%)**														
		Hispanic	37 (42)		20 (51)		17 (49)		.58						
		Black or AA^e^	55 (62)		28 (54)		27 (46)		.93						
		Multiracial or multiethnic	18 (20)		10 (56)		8 (44)		.64						
	BMI-for-age percentile, mean (SD)		74 (26.5)		75 (26.4)		73 (26.8)		.66						
	**BMI category, n (%)**														
		Normal	42 (47)		21 (47)		21 (48)		.91						
		Overweight	19 (21)		9 (20)		10 (23)		—						
		Obese	28 (32)		15 (33)		13 (30)		—						
**Parent**															
	Age (y), mean (SD)		40.83 (8.87)		40.07 (7.67)		41.63 (10.01)		.41						
	**Sex, n (%)**														
		Male	5 (6)		2 (4)		3 (7)		.63						
		Female	84 (94)		43 (96)		41 (93)		—						
	**Race or ethnicity, n (%)**														
		Black or AA	49 (55)		25 (56)		24 (55)		.93						
		Hispanic	40 (45)		22 (49)		18 (51)		.45						
		Multiracial or multiethnic	9 (20)		6 (13)		3 (7)				.31				
	**Country of birth, n (%)**														
		United States	63 (71)		31 (69)		32 (73)				.69				
		Other	26 (29)		14 (31)		12 (27)				—				
	**Bilingual, n (%)**														
		No	62 (70)		31 (69)		31 (71)				.87				
		Yes	27 (30)		14 (31)		13 (30)				—				
	**Education, n (%)**														
		Less than high school	18 (21)		11 (25)		7 (16)				.45				
		Finished high school or GED^f^	10 (11)		6 (14)		4 (9)				—				
		Some college	26 (230)		10 (23)		16 (36)				—				
		Finished college	34 (39)		17 (39)		17 (39)				—				
	**Marital status, n (%)**														
		Single	40 (45)		22 (49)		18 (41)				.70				
		Married or in a marriage-like relationship	35 (39)		17 (38)		18 (41)				—				
		Separated, divorced, or widowed	14 (16)		6 (13)		8 (18)				—				
	**Annual household income (US $), n (%)**														
		<20,000	30 (34)		15 (33)		15 (34)				.66				
		20,000-39,999	30 (34)		17 (38)		13 (30)				—				
		>40,000	29 (33)		13 (29)		16 (36)				—				
	**SNAP^g^ benefits, n (%)**														
		Yes	54 (61)		27 (60)		27 (61)				.90				
		No	35 (39)		18 (40)		17 (39)				—				

^a^E: experimental group.

^b^C: comparison group.

^c^*P* values are based on independent sample *t* tests (continuous variables) or chi-square tests (categorical variables). Conclusions are based on one *P* value, apart from race or ethnicity categories, which are not mutually exclusive.

^d^Not applicable.

^e^AA: African American.

^f^GED: General Educational Development.

^g^SNAP: Supplemental Nutrition Assistance Program.

### Feasibility and Acceptability Measures (Primary Measures)

#### Use of the Web-Based Comic Tool

##### Overview

**Figure 2:**
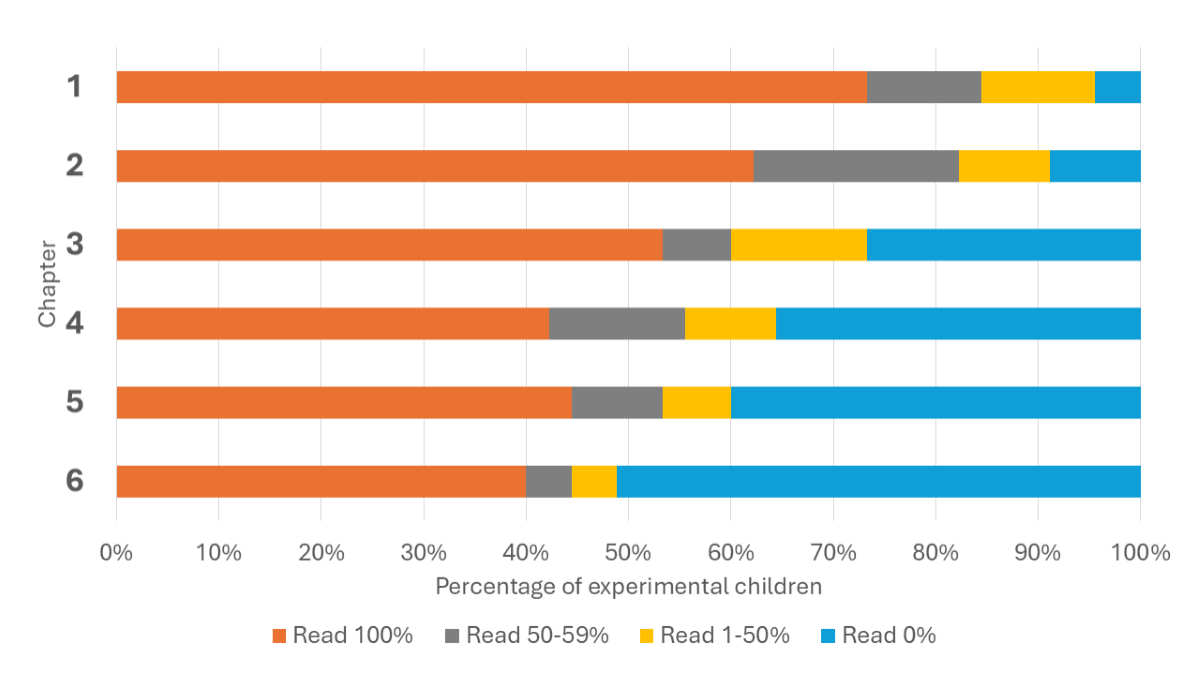
Percentage of the 6 comic chapters that were read by children in the experimental group.

During the 6-week intervention, 73% (33/45) of children in the experimental group accessed their tool weekly. Percentage of those who accessed the tool each week were as follows: week 1, 100% (45/45); week 2, 76% (34/45); week 3, 71% (32/45); week 4, 64% (29/45); week 5, 62% (28/45); and week 6, 56% (25/45). The experimental group accessed the web tool an average of 7.84 (SD 5.09) days, out of 42 possible days. Figure 2 highlights the percentage of children who accessed each of the 6 comic chapters. Of those who accessed the comic chapters, an average of 65% (SD 16.8) of each comic chapter was read, while most of the children (24/44, 53%) read the chapters in full.

In the comparison group, 60% (27/44) accessed their newsletter weekly. Percentage of those who accessed the tool each week were as follows: week 1, 100% (44/44); week 2, 50% (22/44); week 3, 52% (23/44); week 4, 59% (26/44); week 5, 59% (26/44); and week 6, 41% (18/44). Furthermore, the comparison group accessed the web tool an average of 5.82 (SD 4.20) days, out of 42 possible days.

##### Goal Setting Feature

**Figure 3:**
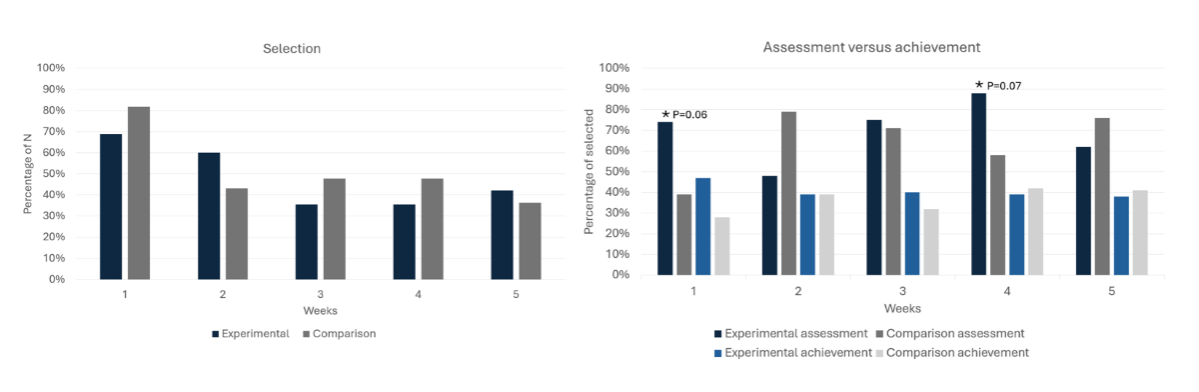
Percentage of experimental and comparison groups who selected, assessed, and reported achieving weekly goals across the 6-week intervention.

On average, 49% (22/45) of children in the experimental group selected a goal each week. Of all the weekly goals that were selected, 67.9% (74/109) were assessed, while 40% (30/74) of those goals were reported to have been achieved. For the comparison group, on average, 51% (23/44) of the children selected a goal. Of all the weekly goals selected, 61.7% (71/115) were assessed. Of the assessed goals, 37% (26/71) were reported to have been achieved. Figure 3 presents the percentage of those in the experimental and comparison groups who selected, assessed, and achieved a goal during each week of the intervention. There was a general decline in goal selection over the weeks in both experimental and comparison groups—in week 1, 69% (31/45) and 82% (36/44) selected goals; by week 5, 42% (19/45) and 36% (16/44) selected goals, respectively. In terms of goal assessment, trends were observed between the experimental and comparison groups in week 1 (*P*=.06) and week 4 (*P*=.07), however, these results were not statistically significant. In week 1, 74% (23/31) and 39% (14/36) assessed goals they selected, while in week 4, 88% (14/16) and 57% (12/21) assessed goals for experimental and comparison groups, respectively (*P*=.07 and *P*=.10, respectively). No other differences were observed across the other weeks. In terms of achieving the weekly goals, the experimental group slightly declined over the weeks (week 1; 11/23, 46% to week 5; 5/12, 42%), while there appeared to be an increase in the comparison group (week 1; 5/14, 36% to week 5; 5/12, 42%).

#### Usability of the Web-Based Comic Tool

Table 4 highlights the mean scores of the 5 usability domains (usability, usefulness, ease of use, ease of learning, and satisfaction) and total for both the experimental and comparison groups. The experimental group reported moderate to high scores in each of the domains. Specifically, ease of learning and ease of use had the highest scores relative to their maximum range (4.58, SD 0.72 and 4.47, SD 0.46, respectively). The mean total score of perceived usability was high and moderately high for the experimental and comparison groups, respectively (4.01, SD 0.37 and 3.81, SD 0.51; *P*=.048).

**Table 4:** Perceived usability of the assigned tool at T3^a^, by group^b^.

Domain	E^c^ (n=43), mean (SD)	C^d^ (n=41), mean (SD)	*P* value
Usability (30 items)	3.14 (0.44)	3.05 (0.40)	—^e^
Usefulness (10 items)	4.35 (0.68)	4.02 (1.11)	—
Ease of use (10 items)	4.47 (0.46)	4.25 (0.66)	—
Ease of learning (2 items)	4.58 (0.72)	4.40 (0.82)	—
Satisfaction (6 items)	4.36 (0.62)	4.07 (0.82)	—
Total	4.01 (0.37)	3.81 (0.51)	.048

^a^T3: intervention end point or 6 weeks postbaseline.

^b^Assessment questionnaire was developed using a total of 30 items from the System Usability Scale [41]; Usefulness, Satisfaction, and Ease-of-use [42] questionnaire; and acceptability or usability measure [43]. Response options ranged from 1 (strongly disagree) to 5 (strongly agree).

^c^E: experimental group.

^d^C: comparison group.

^e^*P* values for individual usability domains have been removed as they were determined to not be applicable.

#### Acceptance and Satisfaction

Overall, children in both arms found their tool to be acceptable, and very few reported any difficulties logging in or accessing content. Specific findings for different components of the comic tool are noted in the following sections.

##### Comic

Most children shared that the comic is a “fun way to read” as it’s exciting, adventurous, motivating, and “helpful like a video game.” Some children explained that the storyline teaches new things about fruits, vegetables, and water and how to stay healthy and take care of oneself. One male child participant described it as “a superhero story where being nutritious is the only way to save the world and junk food slowly starts depleting your energy and, in the end, makes you an awful person.” Most children also reported engaging a parent or family member in their experience of reading the comic.

The comic included a variety of elements aimed at engaging children, including unique comic characters and audio-visual effects. Most children liked the comic characters because of their personalities, which were described as “cool,” “different,” “mysterious,” “admirable” and “brave,” among other adjectives. Some children reported that the characters gave them a good feeling or that they felt a connection to them as they reminded them of someone in their life or shared a favorite hobby. Moreover, nearly all children reported liking the audio or visual effects. Reasons included that the effects made the story more imaginable, “fun to read” and it “gives you a feeling that you are there...in the story.” In addition, some children noted that the audio effects allowed the tool to read for them “if you don’t want to (or can’t) read” and “it’s a pronunciation helper.”

Pop-ups were also used to present additional information related to healthy foods and the characters. Nearly all children liked the pop-ups, describing them as “interesting,” informative,” “surprising,” “cool,” and “pretty funny.” Some children shared that the information pop-ups taught them something they never knew. A male child liked the information pop-ups “because they’re telling stuff that I didn’t really know about; stuff I didn’t know about carrots, I know now that’s why I like it*.*” Some of the reasons shared for liking the character pop-ups included that they helped with developing a deeper understanding of the characters and their role in the story. As 1 male child shared, he “liked [them] because they tell [me] where are they from, what do they like...basically their ideals. It’s like their ID.”

##### Goal Setting

Most children in both groups reported selecting weekly goals both at T2 and T3 time points and acknowledged the “motivation” and “confidence” generated by the process. Some children who did not set weekly goals cited time constraints and involvement in after-school activities as their primary reasons. Most children found the work around goal selection and achievement to be manageable and also easier if the items needed (such as fruit, vegetables, and water) were already available at home and if parental help was accessible to obtain them. Many children also acknowledged that the reminders to work on goals were helpful.

##### Reminders

Most children in both arms preferred text message reminders over email reminders, saying that they were enjoyable to receive, helpful, easier to understand, more convenient, and could be checked more often. In relation to dose and frequency of messaging, across both arms, most children reported receiving “enough messages,” with only a few concluding that they received too many, citing an annoyance at being interrupted or inundated, and preferring a frequency of 2 messages per week.

### Outcome Measures (Secondary Measures)

#### Dietary Beliefs and Attitudes

**Figure 4:**
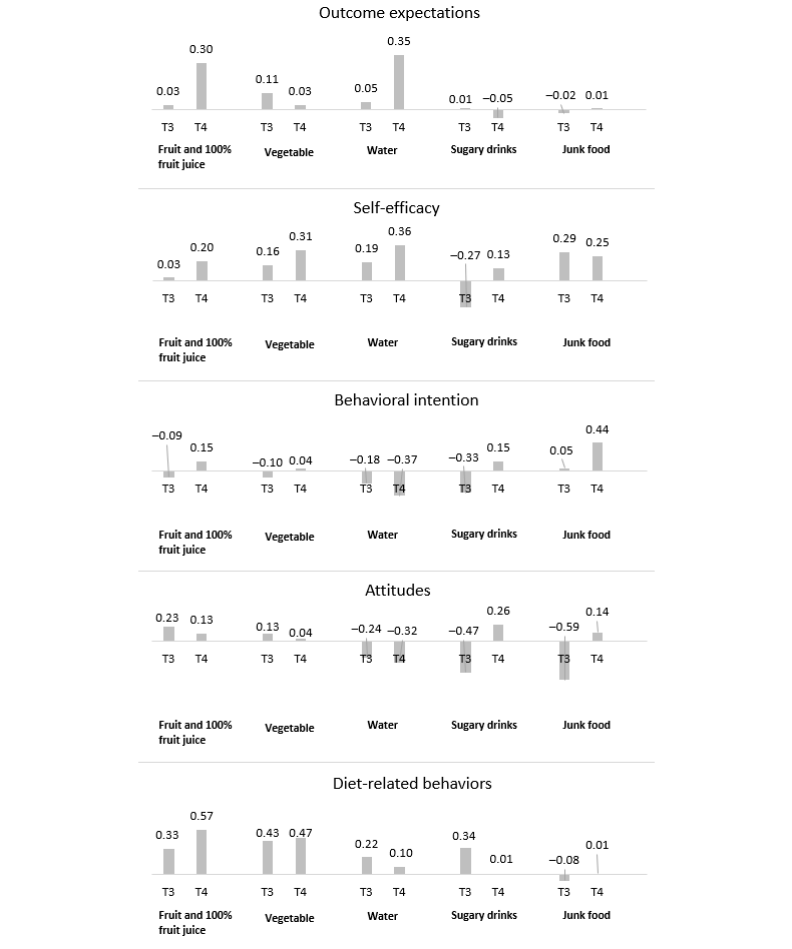
Effect sizes at intervention end or 6 weeks after baseline (T3) and 3-month follow-up after the intervention (T4), comparing experimental (E) and comparison (C) groups on psychosocial variables and self-reported diet-related behaviors. Positive effect size indicates a larger improvement in the E compared to the C group.

At T3, both experimental and comparison groups reported a substantial and consistent increase in OE, self-efficacy, and BI related to fruit and vegetable consumption. In terms of water consumption, both groups reported a substantial increase in BI. The experimental group also reported an increase in self-efficacy for water intake, which was maintained at T4, and the comparison group showed an increase in attitudes toward water consumption. Changes related to OE, self-efficacy, BI, and attitudes toward sugary drinks and junk food consumption were generally small and mixed. In summary, the within-group effect sizes for child dietary beliefs and attitudes were generally larger in the experimental group at T3; however, the majority diminished by T4. The between-group effect sizes, while favoring the experimental group, were in the small range. Refer to Table 5 for the change scores for all psychosocial variables for the experimental and comparison groups and Figure 4 for the effect sizes comparing the experimental and comparison groups. Descriptive statistics by group and child gender are presented in Multimedia Appendix 1.

**Table 5:** Change scores for psychosocial variables related to dietary behaviors from T1^a^ to T3^b^ and T1 to T4^c^, by group.

		Outcome expectations					Self-efficacy					Behavioral intention					Attitudes			
		E^d^ (n=37), mean change (SD)	C^e^ (n=39), mean change (SD)	ES^f,g^ within E	ES within C	E (n=37), mean change (SD)		C (n=39), mean change (SD)	ES within E	ES within C	E (n=37), mean change (SD)		C (n=39), mean change (SD)	ES within E	ES within C	E (n=37), mean change (SD)		C (n=39), mean change (SD)	ES within E	ES within C
**Fruit and 100% fruit juice**																				
	T1 to T3	2.17 (3.43)	2.05 (3.70)	0.63	0.55	0.64 (2.41)		0.59 (1.91)	0.27	0.31	0.45 (1.80)		0.61 (1.61)	0.25	0.38	0.45 (2.49)		–0.13 (2.49)	0.18	–0.05
	T1 to T4	1.00 (3.34)	–0.08 (3.79)	0.30	–0.02	–0.22 (2.78)		–0.74 (2.49)	–0.08	–0.30	0.22 (2.00)		–0.10 (2.19)	0.11	–0.05	–0.36 (2.77)		–0.69 (2.27)	–0.13	–0.30
**Vegetable**																				
	T1 to T3	1.69 (3.21)	1.29 (3.76)	0.53	0.34	1.50 (3.09)		1.02 (2.68)	0.48	0.38	0.60 (2.40)		0.80 (1.96)	0.25	0.41	0.98 (3.20)		0.54 (3.51)	0.30	0.15
	T1 to T4	0.16 (3.96)	0.03 (4.63)	0.04	0.01	0.65 (3.51)		–0.41 (3.30)	0.19	–0.12	0 (1.90)		–0.08 (2.16)	0	–0.04	0.50 (3.01)		0.38 (3.48)	0.17	0.11
**Water**																				
	T1 to T3	1.00 (3.56)	0.78 (5.00)	0.28	0.16	0.31 (1.00)		0.12 (0.95)	0.31	0.13	0.52 (1.80)		0.93 (2.54)	0.29	0.36	0.50 (2.24)		1.17 (3.19)	0.22	0.37
	T1 to T4	0.57 (3.09)	–0.67 (3.91)	0.18	–0.17	0.32 (0.94)		0 (0.83)	0.34	0	–0.16 (1.28)		0.62 (2.62)	–0.13	0.23	–0.33 (2.07)		0.56 (3.26)	–0.16	0.17
**Sugary drinks**																				
	T1 to T3	–0.10 (4.01)	–0.07 (3.29)	–0.02	–0.02	0.21 (2.09)		–0.30 (1.73)	0.10	–0.17	0.55 (2.44)		–0.37 (3.02)	0.22	–0.12	0.10 (3.14)		–1.44 (3.24)	0.03	–0.44
	T1 to T4	–0.22 (3.31)	–0.38 (2.98)	–0.07	–0.13	–0.19 (2.01)		0.08 (2.07)	–0.10	0.04	0.11 (2.17)		0.49 (2.79)	0.05	0.17	–0.27 (2.93)		–0.51 (3.11)	–0.09	0.16
**Junk food**																				
	T1 to T3	0.26 (2.56)	0.32 (3.09)	0.10	0.10	–0.14 (2.42)		0.46 (1.64)	–0.06	0.28	0.40 (2.58)		0.55 (3.31)	0.16	0.17	1.17 (3.01)		–0.63 (2.84)	0.39	–0.22
	T1 to T4	0.43 (2.24)	0.41 (2.60)	0.19	0.16	–0.24 (2.02)		0.18 (1.35)	–0.12	0.13	0.06 (2.62)		1.21 (2.56)	0.02	0.47	0.41 (3.13)		0.77 (2.16)	0.13	0.36

^a^T1: baseline.

^b^T3: intervention end or 6 weeks postbaseline.

^c^T4: 3-month follow-up postintervention.

^d^E: experimental group.

^e^C: comparison group.

^f^ES: effect size.

^g^“ES within” represents changes in score divided by the SD of change within the condition (E and C, respectively); positive ES indicates improvement, except for junk food and sugary drinks, where negative ES indicates improvement.

#### Dietary Behaviors

The experimental group reported a substantial increase in fruit, vegetable, and water intake, which were maintained at T4. Changes in the comparison group were small. Both groups reported a decrease in junk food intake at T3, and the experimental group additionally reported a decrease in sugary drinks intake from T1 to T3; however, these improvements were diminished at T4. The between-group effect sizes for the child dietary intake, favoring the experimental group, were medium to large. Refer to Table 6 for the changes in self-reported diet-related behaviors and Figure 4 for the related effect sizes at T3 and T4 comparing the experimental and comparison groups. Descriptive statistics by child gender are presented in Multimedia Appendix 1.

**Table 6:** Change in self-reported diet-related behaviors from T1^a^ to T3^b^ and T1 to T4^c^, by group.

			E^d^ (n=37), mean change (SD)		C^e^ (n=39), mean change (SD)		ES^f,g^ within E		ES within C
**Fruit and 100% fruit juice**									
	T1 to T3	0.71 (5.12)		–0.80 (4.04)		0.14		–0.20	
	T1 to T4	1.30 (3.39)		–0.79 (3.71)		0.38		–0.21	
**Vegetable**									
	T1 to T3	3.10 (7.27)		0.22 (5.82)		0.43		0.04	
	T1 to T4	3.08 (4.58)		0.51 (5.99)		0.67		0.09	
**Water**									
	T1 to T3	2.52 (6.85)		1.00 (6.76)		0.37		0.15	
	T1 to T4	2.22 (4.84)		1.64 (6.41)		0.46		0.26	
**Sugary drinks**									
	T1 to T3	–1.80 (5.31)		0 (5.34)		–0.34		0	
	T1 to T4	–0.03 (7.06)		0.05 (5.50)		0		0.01	
**Junk food**									
	T1 to T3	–1.55 (4.77)		–1.95 (5.63)		–0.32		–0.35	
	T1 to T4	–1.08 (6.40)		–1.03 (4.91)		–0.17		–0.21	

^a^T1: baseline.

^b^T3: intervention end or 6 weeks postbaseline.

^c^T4: 3-month follow-up postintervention.

^d^E: experimental group.

^e^C: comparison group.

^f^ES: effect size.

^g^“ES within” represents changes in score divided by the SD of change within the condition (E and C); positive ES indicates improvement, except for junk food and sugary drinks, where negative ES indicates improvement.

#### BMI-for-Age Percentile

The mean change between T1 and T4 in BMI-for-age percentile was small and comparable in both experimental and comparison groups (mean –0.58, SD 5.91 and mean –0.50, SD 11.49, respectively). A post hoc exploration by child’s sex showed that BMI-for-age percentile tended to decrease in the experimental group for male participants (mean change –3.26, SD 4.67) and the comparison group for female participants (mean change –3.30, SD 9.77). Refer to Table 7 for BMI-for-age percentile for the experimental and comparison groups, overall and by sex, at T1 and T4.

**Table 7:** BMI-for-age percentile at T1^a^ and T4^b^ for E^c^ and C^d^ groups, overall and among male and female participants.

	Overall		Male participants		Female participants	
	E (n=36)	C (n=36)	E (n=14)	C (n=15)	E (n=22)	C (n=21)
T1, mean (SD)	74.94 (26.01)	70.29 (27.29)	73.74 (29.53)	70.13 (28.82)	75.67 (24.29)	70.40 (26.84)
T4, mean (SD)	73.70 (26.77)	70.57 (29.44)	70.48 (32.12)	73.55 (31.07)	75.76 (23.34)	66.44 (28.81)
T1 to T4, mean change (SD)	–0.58 (5.91)	–0.50 (11.41)	–3.26 (4.67)	3.42 (12.68)	–1.12 (6.09)	–3.30 (9.77)

^a^T1: baseline.

^b^T4: 3-month follow-up postintervention.

^c^E: experimental group.

^d^C: comparison group.

## Discussion

### Principal Findings

The primary purpose of this study was to assess the feasibility and participant acceptability of an interactive, web-based comic tool. In addition, this study explored the tool’s potential impact on dietary behaviors, related psychosocial variables, and BMI-for-age percentile.

There was a decline in use of the digital tool (in both groups) over the 6-week intervention period. While direct comparisons are difficult due to the lack of standardization in how engagement has been defined and measured in previous research [44], low to moderate engagement and a decline in use as an intervention progresses are commonly reported [45-47]. This is concerning given that engagement is critical to the effectiveness of digital health interventions [48]. This was a key consideration during the design process for our tool as we used user-centered design principles throughout the development process [49], with the goal of ultimately developing a culturally relevant and meaningful tool. To enhance potential engagement, we incorporated interactive features into the tool, such as tap or click icons that opened pop-up windows (highlighting health facts, food-related fun facts, or character information, such as a character’s favorite healthy food and beverage).

While engagement did decline in both groups, the use data appear to support the feasibility and acceptability of the comic tool as children in the experimental group were more engaged; an average of 72% (33/45) of the experimental group accessed their tool weekly compared to 60% (27/44) of the comparison group. Furthermore, most of the children in the experimental group, who accessed the comic, actually read the complete chapter (which ranged from 18 to 34 pages) each week. In addition to a higher level of engagement, the experimental group also reported higher usability of their tool, which is an indicator of tool acceptability and satisfaction [50,51].

Other interactive features included embedded audio recordings for certain character dialogue, which also presented opportunities to deliver health messages in another format. Such a format can help with the understanding of the health messages promoted within the comic [52], particularly as low literacy levels continue to exist in our intended population [53-55]. Research has found that Black and Hispanic students begin high school with literacy skills 3 years behind those of White and Asian students [53]. In addition, students from families with low incomes enter high school with literacy skills that are 5 years behind those of students from families with high incomes [55]. Feedback from participants acknowledged the benefits of the embedded interactive features as 1 child noted that the audio effects allow the tool to read for the children, “if you don’t want to or can’t read.” Such features can also add to the immersion of the reader into the story, as studies have found that audio features can improve the manga reading experience provided it connects the reader with the objects represented in the scene [56]. This could lead to enhanced persuasion of the story’s health messages and, thus, motivate positive behavior change, as suggested by the Narrative Transportation Theory [57,58].

While several BCTs were incorporated into the digital comic tool, a key component of the tool was the goal selection and assessment activity. Research has shown that the BCT of goal setting is associated with positive health outcomes, although primarily related to BMI measures in children above a healthy weight [59]. Children in our study acknowledged that goal achievement was easier if the appropriate support was in place, which included fruit, vegetables, and water being available at home and if parental help was accessible. This highlights the importance of ensuring that healthy foods are accessible to allow the children to be empowered to make positive choices. It also reinforces the importance of engaging parents to not only be positive role models but to also provide a supportive home environment as parent-directed interventions can improve the self-efficacy of their children [60]. Prompts and cues were also included in the comic tool in the form of 4 reminder messages each week from the characters themselves. The frequency of the reminder messages was informed by formative research with our intended population, as the literature is quite limited in this area [61]. While a few children did note that they received too many, overall, the children acknowledged that the reminder function, comprising 4 texting or email messages each week was “enough” and an important feature to promote sustained engagement [61].

Secondary outcomes of this study included changes in psychosocial variables related to specific diet-related behaviors, dietary intake, and BMI-for-age percentile. Results showed that while changes in psychosocial variables for certain diet-related behaviors were observed in both the experimental and comparison groups from T1 to T3, most improvements were diminished by T4. However, improvements in intake related to fruit, vegetable, and water behaviors observed in the experimental group were maintained at T4. Furthermore, while no differences were observed in BMI-for-age percentile change between both groups from T1 to T4, a post hoc analysis found that BMI percentile tended to decrease in the experimental group for male participants and in the comparison group for female participants. The improvement in fruit, vegetable, and water intake that was maintained through the T4 period in the experimental group may partially explain the anthropometric findings observed in the male participants, as such behaviors are recommended to reduce obesity risk [62,63]. Further research is warranted to better understand the mechanisms that may have resulted in the observed trend of the decreased BMI percentile in both groups, particularly as no sustained changes were found in related psychosocial variables and no significant improvements in dietary behaviors were observed in the comparison group.

Sex differences in the BMI percentile outcome are interesting to note. Health-promoting comics have been used effectively to reach children of both genders [41,64,65]; however, in our study, it appears that male participants may have responded more positively to the comic tool. While extensive formative research was conducted with both genders from our intended population, with the goal of developing a broad-reaching, meaningful, and engaging narrative, the main character of the story was male. This may have led to unintended differences in how male and female participants responded to the health messages. Interestingly, prior research by the team found that both male and female children often selected a male character when asked who their favorite comic character was [42]; however, other research has shown that gender does play a role in how people relate and respond to media characters [43,66]. Furthermore, while we aimed to address a broad range of genres in the comic to appeal to both genders, differences in genre preferences do exist between the sexes as females tend to prefer romance, fantasy, and action, while males tend to prefer humor and action when reading comics [67].

### Implications for Future Research

Further research is needed to not only understand the elements that influence tool engagement but to also gain a better understanding of the comic tool’s key components that influenced the observed health-related outcomes. This pilot and feasibility study suggests that such a comic tool could be an appealing format to promote positive beliefs and behaviors related to a healthier diet in racial and ethnic minority preadolescents (particularly in male children). In addition, our study had successful recruitment and high retention with our participants [23]. Furthermore, 81% (61/75) of the children reported at T4 that they were very satisfied or extremely satisfied with their interactions with the study staff [23]. Therefore, a full-scale RCT with a longer follow-up period is warranted to determine the effectiveness of Intervention INC. However, given potential gender differences in character relatability and genre preferences, the development of future health-promoting comic tools should more carefully consider how to frame storylines and messaging to effectively impact both genders. Moreover, as newly adopted behaviors often diminish once an intervention ends [68], a maintenance intervention should be considered following the initial intervention period, as it could provide critical continued support and reinforce newly adopted behaviors. In addition, this tool’s messages mainly focused on obesity-related dietary behaviors. As physical activity is a key contributing factor to reducing obesity risk, the inclusion of a physical activity component should be considered to produce a more comprehensive tool focused on additional influences related to childhood obesity.

### Limitations

It is important to acknowledge that this study is not without its limitations. We were unable to capture more granular use data, such as the minutes participants used the tool itself. Self-report data are always a challenge, in particular with dietary intake data. This limitation is further exacerbated by the fact that our questionnaires, while informed by validated questionnaires commonly used in the literature [25-30], were not validated tools and were completed by the children themselves. However, it should be acknowledged that T1 and T4 data were collected in person where a research team member was present and available to answer any questions and assist the children in completing the questionnaire. Seasonality issues may be a concern with the outcome measures, as recruitment was conducted on a rolling basis over a 3-month period. Thus, some participants completed the study during the fall season, while others completed it during the spring season. Another limitation is the small sample size, thus limiting statistical power. The generalizability of these findings is also limited as the data are unique to racial and ethnic minority preadolescent children residing in underserved NYC communities. In addition, all study participants had to have ongoing access to the internet, further limiting the study’s generalizability.

### Conclusions

This pilot and feasibility study suggests that the multimedia platform of comics could be an appealing and engaging format to promote healthy diet-related beliefs and behaviors in racial and ethnic minority, urban preadolescents at risk of childhood obesity. Furthermore, as comics are popular among US youth and can be easily disseminated, particularly in digital format, the graphics and minimal text make it a promising format for low-literacy populations. The sex differences observed in BMI percentile suggest that boys may respond more positively to health messages delivered in such a format; however, the comic’s main character (who is male) may have been more relatable to our male participants. Thus, future iterations of the comic should carefully consider the main character to ensure they are relatable to all genders. While the results are promising, further research is needed, including a full-scale RCT, to determine the effectiveness of Intervention INC.

## Data Availability

The datasets generated and analyzed during this study are available from the corresponding author on reasonable request.

## Appendix

Multimedia Appendix 1 Descriptive statistics for psychosocial variables and self-reported diet related behaviors across timepoints, by group and by sex.

Multimedia Appendix 2 CONSORT-eHEALTH checklist (V 1.6.2).

## Abbreviations

BCT: behavior change technique
BI: behavioral intention
CONSORT: Consolidated Standards of Reporting Trials
INC: Interactive Nutrition Comics for Urban, Minority Preadolescents
NYC: New York City
OE: outcome expectation
RCT: randomized controlled trial
T1: baseline
T2: intervention midpoint or 3 weeks after baseline
T3: intervention end or 6 weeks after baseline
T4: 3-month follow-up
