# Supplementary figures and images for: Testing a Web-Based Interactive Comic Tool to Decrease Obesity Risk Among Racial and Ethnic Minority Preadolescents: Randomized Controlled Trial
Source: JMIR Form Res. 2025 Jan 15;9:e58460. doi: 10.2196/58460 (PMC11780287; doi:10.2196/58460)

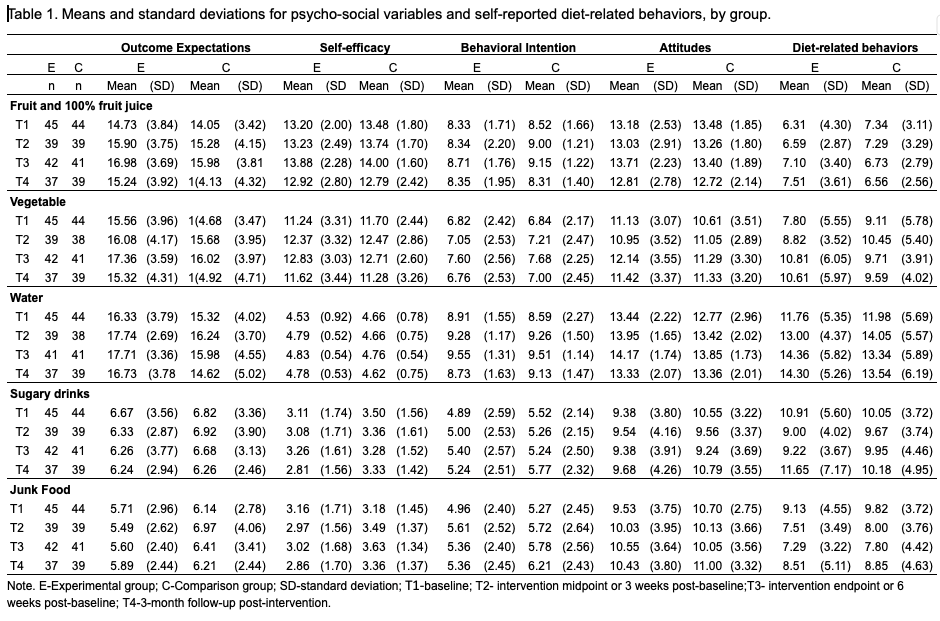


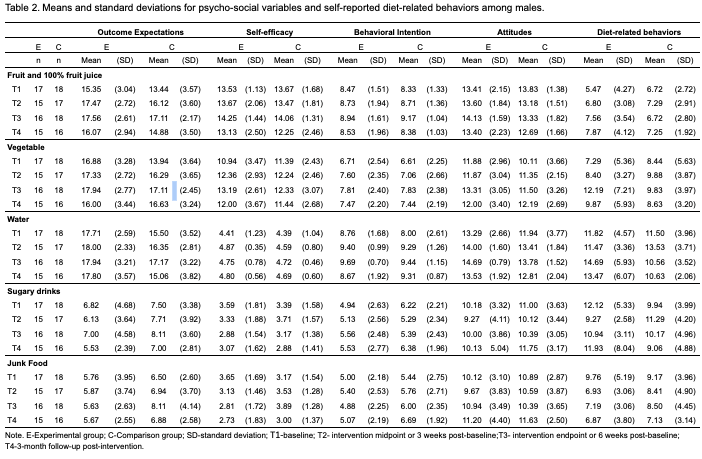


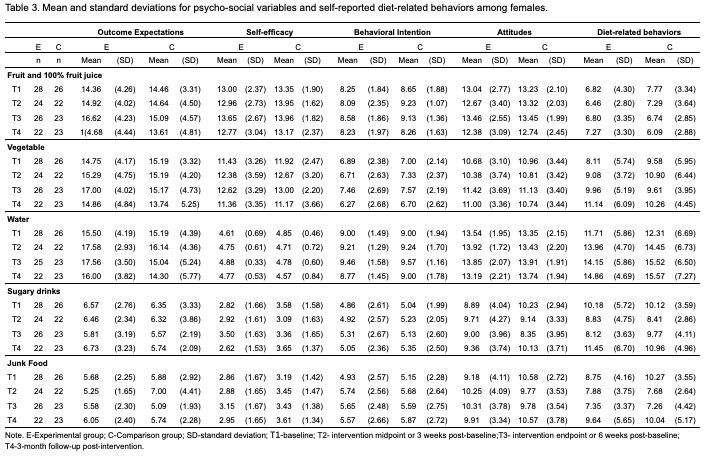


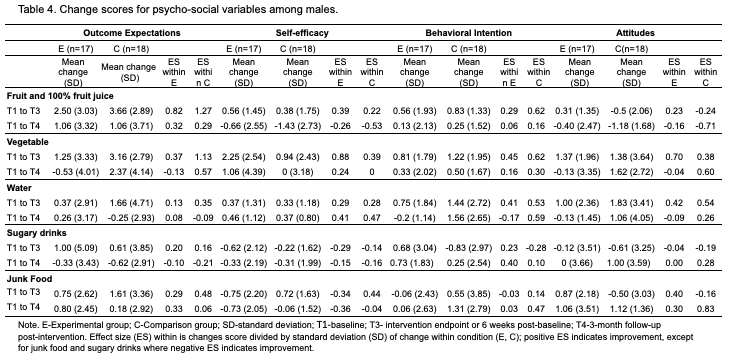


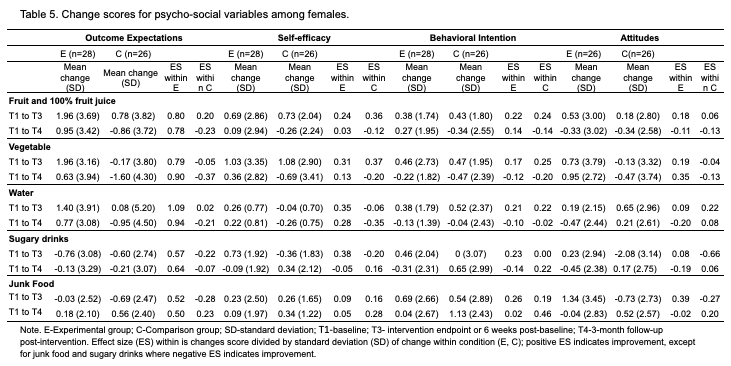

Supplement: Multimedia Appendix 1 [file formative_v9i1e58460_app1.docx]
