# Supplementary material for: Testing a Web-Based Interactive Comic Tool to Decrease Obesity Risk Among Racial and Ethnic Minority Preadolescents: Randomized Controlled Trial
Source: JMIR Form Res. 2025 Jan 15;9:e58460. doi: 10.2196/58460 (PMC11780287; doi:10.2196/58460)
Supplement: Multimedia Appendix 2 [file formative_v9i1e58460_app2.pdf]

CONSORT-EHEALTH Checklist V1.6.2 Report	Manuscript Number	58460

(based on CONSORT-EHEALTH V1.6), available at [http://tinyurl.com/consort-ehealth-v1-6].

Date completed

4/6/2024 17:04:42

by

May May

TITLE

1a-i) Identify the mode of delivery in the title

1a-ii) Non-web-based components or important co-interventions in title

Yes it does. Title includes “Web-based”

1a-iii) Primary condition or target group in the title

ABSTRACT

1b-i) Key features/functionalities/components of the intervention and comparator in the METHODS section of the ABSTRACT

1b-ii) Level of human involvement in the METHODS section of the ABSTRACT

“a pilot two-group randomized study, comprising a 6-week intervention and a 3-month follow-up period. Participants were randomly assigned to the experimental (E) (n=45) group, who received the web-based comic tool, or the comparison (C) (n=44) group, who received online nutrition newsletters.” Intervention details published - JMIR Res Protoc 2018;7(11):e10682

1b-iii) Open vs. closed, web-based (self-assessment) vs. face-to-face assessments in the METHODS section of the ABSTRACT

1b-iv) RESULTS section in abstract must contain use data

1b-v) CONCLUSIONS/DISCUSSION in abstract for negative trials

INTRODUCTION

2a-i) Problem and the type of system/solution

2a-ii) Scientific background, rationale: What is known about the (type of) system

“However, knowledge gaps in the usability, engagement, and usefulness of such applications still exist, limiting their potential effectiveness.16 Furthermore, there is a lack of effective health promotion interventions that have been culturally-tailored to meet the needs and preferences of populations with disproportionate rates of chronic disease; and even fewer have been developed for children, and in particular those transitioning from childhood to adolescence.17 It is crucial to intervene during this development stage as food preferences established during this period often persist into adulthood.18–20 Additionally, children at this age are gaining autonomy and developing decision-making skills related to dietary behaviors,21 which highlight the importance of intervening at this critical period.”

METHODS

3a) CONSORT: Description of trial design (such as parallel, factorial) including allocation ratio

3b) CONSORT: Important changes to methods after trial commencement (such as eligibility criteria), with reasons

“to assess the feasibility and participant acceptability of Intervention INC, an interactive, Web-based health promotion tool."

3b-i) Bug fixes, Downtimes, Content Changes

4a) CONSORT: Eligibility criteria for participants

“The Intervention INC study was a pilot, two-group randomized controlled trial (RCT) that evaluated a six-week intervention promoting fruit, vegetable (F/V) and water consumption, with a three-month follow-up period.” A protocol was published/registered with the Clinical Trials Registry (NCT03165474).

4a-i) Computer / Internet literacy

4a-ii) Open vs. closed, web-based vs. face-to-face assessments:

4a-iii) Information giving during recruitment

“Multiple recruitment approaches utilizing bilingual materials, such as flyering, attending community events and sending mailings to parents/guardians of potentially eligible child patients of a community-based clinic in NYC, were implemented between August and November 2017."

4b) CONSORT: Settings and locations where the data were collected

Eligibility criteria was modified, but noted in prior ms - JMIR Res Protoc 2018;7(11):e10682.

4b-i) Report if outcomes were (self-)assessed through online questionnaires

4b-ii) Report how institutional affiliations are displayed

”T1 and T4 data collection was conducted in-person at the study site, while T2 and T3 data collection was conducted via phone. At T2 and T3, links to any questionnaires were sent to the participants ahead of time to complete and then responses were confirmed during the phone call. If the questionnaire was not completed prior to the call, the child completed it during the call with a trained research assistant.”
“Self-reported data from both the children and parents have been collected, which pose a validity risk, due to intentional/unintentional misreporting”

5) CONSORT: Describe the interventions for each group with sufficient details to allow replication, including how and when they were actually administered

5-i) Mention names, credential, affiliations of the developers, sponsors, and owners

5-ii) Describe the history/development process

5-iii) Revisions and updating

5-iv) Quality assurance methods

5-v) Ensure replicability by publishing the source code, and/or providing screenshots/screen-capture video, and/or providing flowcharts of the algorithms used

5-vi) Digital preservation

5-vii) Access

5-viii) Mode of delivery, features/functionalities/components of the intervention and comparator, and the theoretical framework

”Children received up to US $70 and parents/guardians received up to US $65 (in the form of gift cards) for completing data collection."

5-ix) Describe use parameters

”Children in the E group received access to Intervention INC (Interactive Nutrition Comics for Urban, Minority Preadolescents), a theory-informed interactive Web-based comic tool, which comprises a six-chapter comic with pop-up windows (highlighting health facts, food-related fun facts, or character information) and prompted audio/visual effects to enhance tool engagement. It also includes a weekly goal setting and assessment feature and text/email messages reminding the child to read the comic and work on their selected health behavior goals. The comic tool was designed and developed by a collaborative research team, consisting of scientists, digital health experts, content and system developers, and intended users (Black/AA and Hispanic preadolescents and their parents). Extensive formative research, including usability testing, was conducted during the development process."		
**5-x) Clarify the level of human involvement**		
**5-xi) Report any prompts/reminders used**		
**5-xii) Describe any co-interventions (incl. training/support)**		
“It also includes a weekly goal setting and assessment feature and text/email messages reminding the child to read the comic and work on their selected health behavior goals.” "Similar to the E group, the C group also had a weekly goal setting and assessment component and received text/email reminder messages.”		
**6a) CONSORT: Completely defined pre-specified primary and secondary outcome measures, including how and when they were assessed**		
“Children residing in New York City (NYC) who self-identified as Black/AA and/or Hispanic, aged nine to 12 years at the time of baseline visit, read/spoke English, had a body mass index (BMI) percentile at or above 5% at baseline (categorized as healthy, overweight, or obese) were recruited. Parents/guardians of a child willing to participate in the study, who read/spoke English or Spanish, and had primary responsibility for preparing/purchasing food for the child were recruited as well. Additional inclusionary/exclusionary criteria were considered and described in a previous publication by Leung et al.22		
**6a-i) Online questionnaires: describe if they were validated for online use and apply CHERRIES items to describe how the questionnaires were designed/deployed**		
**6a-ii) Describe whether and how “use” (including intensity of use/dosage) was defined/measured/monitored**		
**6a-iii) Describe whether, how, and when qualitative feedback from participants was obtained**		
**6b) CONSORT: Any changes to trial outcomes after the trial commenced, with reasons**		
“T1 and T4 data collection was conducted in-person at the study site, while T2 and T3 data collection was conducted via phone. At T2 and T3, links to any questionnaires were sent to the participants ahead of time to complete and then responses were confirmed during the phone call. If the questionnaire was not completed prior to the call, the child completed it during the call with a trained research assistant.”		
**7a) CONSORT: How sample size was determined**		
**7a-i) Describe whether and how expected attrition was taken into account when calculating the sample size**		
**7b) CONSORT: When applicable, explanation of any interim analyses and stopping guidelines**		
“Data Collection and Measures<br>Data were collected at four different time points: baseline (T1), intervention midpoint or three weeks post-baseline (T2), intervention endpoint or six weeks post-baseline (T3), and three-month follow-up post-intervention (T4). Below includes brief descriptions of our primary and secondary outcome measures as details of all measures are described elsewhere.22 T1 and T4 data collection was conducted in-person at the study site, while T2 and T3 data collection was conducted via phone. At T2 and T3, links to any questionnaires were sent to the participants ahead of time to complete and then responses were confirmed during the phone call. If the questionnaire was not completed prior to the call, the child completed it during the call with a trained research assistant.<br>Further details are including in current ms and previously published ms. However, the form limited response length.		
**8a) CONSORT: Method used to generate the random allocation sequence**		
No changes in trial outcomes after trial commenced.		
**8b) CONSORT: Type of randomisation; details of any restriction (such as blocking and block size)**		
This question is not applicable.		
**9) CONSORT: Mechanism used to implement the random allocation sequence (such as sequentially numbered containers), describing any steps taken to conceal the sequence until interventions were assigned**		
This question is not applicable. Please see 8b.		
**10) CONSORT: Who generated the random allocation sequence, who enrolled participants, and who assigned participants to interventions**		
“Parent-child dyads were randomized into either the experimental (E) or comparison (C) group, with both child and parent participants blinded to their assigned group. Details regarding the randomization process are described in the protocol paper.” - Testing a Web-Based Interactive Comic Tool to Decrease Obesity Risk Among Minority Preadolescents: Protocol for a Pilot Randomized Control Trial JMIR Res Protoc 2018;7(11):e10682		
**11a) CONSORT: Blinding - If done, who was blinded after assignment to interventions (for example, participants, care providers, those assessing outcomes) and how**		
**11a-i) Specify who was blinded, and who wasn’t**		
**11a-ii) Discuss e.g., whether participants knew which intervention was the “intervention of interest” and which one was the “comparator”**		
“Parent-child dyads were randomized into either the experimental (E) or comparison (C) group, with both child and parent participants blinded to their assigned group.”<br>“Of note, the data analyst was blinded to intervention assignment to minimize bias.”		
**11b) CONSORT: If relevant, description of the similarity of interventions**		
“Parent-child dyads were randomized into either the experimental (E) or comparison (C) group, with both child and parent participants blinded to their assigned group. Details regarding the randomization process are described in the protocol paper.”<br>JMIR Res Protoc 2018;7(11):e10682.		
**12a) CONSORT: Statistical methods used to compare groups for primary and secondary outcomes**		
JMIR Res Protoc 2018;7(11):e10682		
**12a-i) Imputation techniques to deal with attrition / missing values**		
**12b) CONSORT: Methods for additional analyses, such as subgroup analyses and adjusted analyses**		
”Children in the C group received access to six Web-based newsletters. Health-related content for the C group was similar to that for the E group but presented in a newsletter (didactic) format, which includes tips, recipes, diet-related knowledge/facts, and online games. Similar to the E group, the C group also had a weekly goal setting and assessment component and received text/email reminder messages.”		
RESULTS		
**13a) CONSORT: For each group, the numbers of participants who were randomly assigned, received intended treatment, and were analysed for the primary outcome**		
**13b) CONSORT: For each group, losses and exclusions after randomisation, together with reasons**		
Due to the pilot nature of this study, the analyses were not adjusted for inflated Type I error due to testing multiple hypotheses.		
**13b-i) Attrition diagram**		
**14a) CONSORT: Dates defining the periods of recruitment and follow-up**		
“A total of 89 child-parent dyads were recruited and completed baseline measures.” Participants were randomly assigned to the experimental (E) (n=45) group or the comparison (C) (n=44) group.		
**14a-i) Indicate if critical “secular events” fell into the study period**		
**14b) CONSORT: Why the trial ended or was stopped (early)**		

This was not noted in the current ms as a CONSORT flow diagram was published in - Testing a Web-Based Interactive Comic Tool to Decrease Obesity Risk Among Minority<br>Preadolescents: Protocol for a Pilot Randomized Control Trial JMIR Res Protoc 2018;7(11):e10682		
**15) CONSORT: A table showing baseline demographic and clinical characteristics for each group**		
“Multiple recruitment approaches utilizing bilingual materials, such as flyering, attending community events and sending mailings to parents/guardians of potentially eligible child patients of a community-based clinic in NYC, were implemented between August and November 2017.”		
**15-i) Report demographics associated with digital divide issues**		
**16a) CONSORT: For each group, number of participants (denominator) included in each analysis and whether the analysis was by original assigned groups**		
**16-i) Report multiple “denominators” and provide definitions**		
**16-ii) Primary analysis should be intent-to-treat**		
Sample sizes for each analysis are indicated in the ms tables. Effect sizes are reported for all relevant outcomes.		
**17a) CONSORT: For each primary and secondary outcome, results for each group, and the estimated effect size and its precision (such as 95% confidence interval)**		
Not applicable as the trial was not stopped early.		
**17a-i) Presentation of process outcomes such as metrics of use and intensity of use**		
**17b) CONSORT: For binary outcomes, presentation of both absolute and relative effect sizes is recommended**		
“Table 1. Baseline child and parent demographic characteristics” provide these data.		
**18) CONSORT: Results of any other analyses performed, including subgroup analyses and adjusted analyses, distinguishing pre-specified from exploratory**		
All results for this pilot study are reported using summary statistics, including standard deviations, as well as effect sizes.		
**18-i) Subgroup analysis of comparing only users**		
**19) CONSORT: All important harms or unintended effects in each group**		
This question is not applicable.		
**19-i) Include privacy breaches, technical problems**		
**19-ii) Include qualitative feedback from participants or observations from staff/researchers**		
DISCUSSION		
**20) CONSORT: Trial limitations, addressing sources of potential bias, imprecision, multiplicity of analyses**		
**20-i) Typical limitations in ehealth trials**		
**21) CONSORT: Generalisability (external validity, applicability) of the trial findings**		
**21-i) Generalizability to other populations**		
**21-ii) Discuss if there were elements in the RCT that would be different in a routine application setting**		
**22) CONSORT: Interpretation consistent with results, balancing benefits and harms, and considering other relevant evidence**		
**22-i) Restate study questions and summarize the answers suggested by the data, starting with primary outcomes and process outcomes (use)**		
**22-ii) Highlight unanswered new questions, suggest future research**		
"The primary purpose of this study was to assess the feasibility and participant acceptability of an interactive, Web-based comic tool. In addition, this study explored the tool’s potential impact on dietary behaviors, related psycho-social variables and BMI-for-age %ile. The usage data collected support the feasibility and acceptability of the comic tool as children in the E group appeared to be more engaged (as defined by greater tool access). Further, the majority of the children in the E group, who accessed the comic, actually read the complete chapter (which ranged from 18 to 34 pages) each week. In addition to a higher level of engagement, the E group also reported higher total usability of their tool. Usability testing is helpful to not only assess tool acceptability and satisfaction, but can also inform how to enhance a tool’s usefulness and potential effectiveness for end users.38,39 These usability results are consistent with those found in a previous study by our research team, which further support the utilization of a narrative approach to promoting positive health behaviors in the form of comics, where health messaging is embedded into the storyline and interactive features are incorporated to enhance engagement.24"		
Other information		
**23) CONSORT: Registration number and name of trial registry**		
We conducted one not prespecified exploratory analysis on the BMI changes by gender.<br>“BMI-for-age Percentile<br>The mean change between T1 and T4 in BMI-for-age %ile was small and comparable in both E and C groups (-.58 ± 5.91 and -.50 ± 11.49, respectively). These improvements were not statistically significant (p>.05). A post hoc exploration by child’s sex showed that BMI-for-age %ile tended to decrease in E group for males (mean change -3.26 ± 4.67) and C group for females (mean change -3.30 ± 9.77).”		
**24) CONSORT: Where the full trial protocol can be accessed, if available**		
This ms does not mention any important harms or unintended effects in either group. However, no harms or unintended effects occurred within our study participants.		
**25) CONSORT: Sources of funding and other support (such as supply of drugs), role of funders**		
“Trial Registration: NCT03165474” at Clinicaltrials.gov		
**X26-i) Comment on ethics committee approval**		
**x26-ii) Outline informed consent procedures**		
**X26-iii) Safety and security procedures**		
**X27-i) State the relation of the study team towards the system being evaluated**		
